# Minimally Invasive Implant Rehabilitation in Geriatric Patients: A Comprehensive Narrative Review of Contemporary Strategies to Avoid Extensive Bone Augmentation Procedures

**DOI:** 10.3390/geriatrics11050117

**Published:** 2026-09-01

**Authors:** Angelo Aliberti, Wien Cirine Ben Amor, Mauro Mariniello, Gilberto Sammartino, Oreste Trosino, Francesco Giordano

**Affiliations:** 1 Department of Neuroscience, Reproductive Science and Odontostomatological Sciences, University of Naples Federico II, Via Sergio Pansini, 5, 80138 Naples, Italy; ange.aliberti@studenti.unina.it; 2 Research Laboratory Oral Health and Rehabilitation, LR12ES11, Faculty of Dental Medicine, University of Monastir, Monastir 5000, Tunisia; wiemcirine.benamor@hotmail.com; 3 Interdepartmental Research Centre in Health Management and Innovation in Healthcare (CIRMIS), University of Naples Federico II, Via Sergio Pansini, 5, 80138 Naples, Italy; 4 Department of Integrated Time-Dependent Network Activities for Stroke, Surgical Emergencies, and Trauma, Head and Neck Outpatient and Inpatient Specialties, Federico II University Hospital, Via Sergio Pansini 5, 80138 Naples, Italy; trosino@libero.it; 5 Department of Medicine, Surgery and Dentistry Scuola Medica Salernitana, University of Salerno, 84081 Baronissi, Italy; frgiordano@unisa.it

**Keywords:** geriatric dentistry, elderly patients, dental implants, edentulism, alveolar bone atrophy, short implants, bone augmentation, frailty, implant-supported prostheses

## Abstract

**Background/Objectives**: The management of edentulism and severe alveolar bone loss in older adults remains a significant challenge in implant dentistry. Given the potential biological and therapeutic burden associated with extensive regenerative procedures, increasing interest has been directed toward treatment strategies that utilize available anatomy and reduce surgical complexity. This narrative review examined contemporary augmentation-avoidance approaches for implant rehabilitation in geriatric patients. **Methods**: A structured narrative literature search was performed in PubMed/MEDLINE, Scopus, and Web of Science, including studies published up to 30 May 2026. Clinical studies, systematic, umbrella and narrative reviews, meta-analyses that evaluate augmentation-avoidance approaches in implant dentistry were considered. The review examined evidence relevant to older adults, including clinical, prosthetic, functional, and patient-centered outcomes. **Results**: The available literature describes multiple strategies for managing reduced bone availability, including short and ultra-short implants, narrow-diameter implants, tilted implant concepts, pterygoid and trans-sinus implants, zygomatic implants, immediate loading protocols, flapless and computer-guided surgery, and implant-retained overdentures. These approaches have broadened the range of treatment options available for patients with reduced bone availability and may reduce the need for extensive regenerative procedures in selected clinical situations. In addition to implant and prosthetic outcomes, increasing attention has been directed toward oral health-related quality of life, masticatory function, treatment satisfaction, and maintenance requirements. However, evidence specifically focused on geriatric populations remains limited. **Conclusions**: Contemporary implant dentistry offers several alternatives to extensive bone regeneration in carefully selected geriatric patients, although current evidence is largely extrapolated from mixed adult populations. Treatment planning should integrate anatomical conditions with systemic health, functional status, frailty-related factors, and long-term maintenance considerations. Further geriatric-focused research is needed to support patient-centered decision-making and optimize long-term rehabilitation outcomes in aging populations.

## 1. Introduction

Population aging represents one of the most significant demographic transformations of the twenty-first century and is expected to profoundly influence healthcare systems worldwide in the coming decades [1]. Advances in medical care, public health initiatives, and socioeconomic development have contributed to a substantial increase in life expectancy, resulting in a rapidly expanding population of older adults [2]. As longevity continues to rise, preserving functional independence, well-being, and quality of life has become a primary objective of modern healthcare [3]. Within this context, oral health is increasingly recognized as an essential component of healthy aging, extending far beyond the traditional concept of maintaining teeth and oral tissues. Recent research has further emphasized the importance of preventive and restorative strategies specifically tailored to older adults, highlighting the growing interest in age-oriented approaches aimed at preserving oral health and function throughout the aging process [4,5].

The ability to chew efficiently, communicate effectively, and engage confidently in social interactions contributes substantially to overall health and well-being. Oral function plays a critical role in nutritional status, psychological health, and social participation, all of which are particularly relevant in later life [2]. Despite considerable improvements in preventive dentistry and restorative care, partial and complete edentulism remain highly prevalent among older adults worldwide [6]. Tooth loss continues to be associated with periodontal disease, extensive restorative histories, chronic systemic conditions, and disparities in access to dental care [7]. The consequences of edentulism frequently extend beyond the oral cavity, affecting masticatory performance, dietary choices, phonetics, facial esthetics, self-esteem, and oral health-related quality of life [8]. As a result, the demand for effective and durable oral rehabilitation strategies among older individuals continues to increase.

The development of implant dentistry has dramatically transformed the rehabilitation of edentulous and partially edentulous patients [9]. Implant-supported prostheses have become a predictable and widely accepted treatment option capable of restoring function, improving prosthetic stability, and enhancing patient satisfaction across a broad spectrum of clinical situations [10]. Long-term clinical evidence has demonstrated favorable outcomes and high implant survival rates even among elderly individuals, supporting the concept that chronological age alone should not be regarded as a contraindication to implant therapy [11]. Consequently, increasing numbers of older adults seek implant-supported rehabilitation as a means of improving comfort, oral function, and quality of life. Nevertheless, implant rehabilitation in geriatric patients presents unique challenges that extend beyond the replacement of missing teeth. Aging is frequently accompanied by complex biological, medical, functional, and psychosocial changes that may directly influence treatment planning and clinical decision-making [12]. The prevalence of chronic conditions such as cardiovascular disease, diabetes mellitus, osteoporosis, and neurodegenerative disorders increases substantially with age, often resulting in more complex clinical scenarios [13,14]. In addition, polypharmacy, functional limitations, cognitive impairment, and varying degrees of dependency may further complicate treatment delivery and long-term maintenance [15]. Although chronological age is frequently used to define older populations, treatment planning in implant dentistry is increasingly influenced by biological and functional age rather than age alone. Older adults represent a highly heterogeneous population in whom frailty, multimorbidity, polypharmacy, cognitive status, functional independence, and self-care capacity may have a greater impact on treatment complexity and long-term outcomes than chronological age itself [16].

Beyond chronological age, frailty has emerged as one of the most relevant determinants of health outcomes in older adults. Frailty is generally characterized by a reduction in physiological reserve and an increased vulnerability to external stressors, resulting in a diminished capacity to maintain homeostasis when exposed to illness, surgery, or other challenging conditions [16]. From a clinical perspective, frailty may significantly influence treatment tolerance, postoperative recovery, maintenance capability, and the overall risk-benefit profile of complex implant interventions. Consequently, treatment planning in elderly patients requires a broader perspective that extends beyond conventional surgical and prosthetic considerations and incorporates an individualized evaluation of the patient’s overall health status, functional capacity, and personal expectations [17].

Anatomical limitations frequently represent an additional challenge in geriatric implant rehabilitation. Long-standing edentulism is commonly associated with progressive alveolar bone resorption, often resulting in reduced bone volume and unfavorable anatomical conditions for conventional implant placement [18]. Historically, severe ridge atrophy has been managed through a variety of bone augmentation procedures aimed at reconstructing deficient alveolar structures before implant insertion [19]. Guided bone regeneration, sinus floor elevation, block grafting, and vertical ridge augmentation have expanded the indications for implant therapy and remain valuable treatment options in contemporary implant dentistry [20]. Long-term clinical evidence has demonstrated the predictability of guided bone regeneration procedures in the management of atrophic ridges [21]. However, these procedures are often associated with increased surgical complexity, prolonged treatment times, higher financial costs, greater patient discomfort, and a higher incidence of biological and technical complications [22]. While such approaches may provide predictable outcomes in appropriately selected patients, their application in elderly individuals requires careful consideration of whether the anticipated benefits justify the additional surgical burden. Nevertheless, favorable outcomes have also been reported for selected regenerative approaches combined with immediate full-arch rehabilitation protocols [23].

Over the past decade, implant dentistry has progressively evolved toward treatment philosophies that prioritize simplification, reduced invasiveness, and the preservation of existing anatomical resources whenever possible [24]. Rather than focusing exclusively on reconstructing lost tissues, contemporary clinicians increasingly seek therapeutic strategies capable of maximizing the use of available native bone while minimizing treatment morbidity [25]. This evolution has been facilitated by substantial advances in implant design, implant surface technology, digital planning, computer-guided surgery, and prosthetic protocols [26]. Therefore, a growing number of treatment options are now available for patients presenting with reduced bone volume, frequently allowing clinicians to avoid extensive bone augmentation procedures without compromising functional outcomes. Although augmentation-avoidance strategies often reduce the need for reconstructive procedures, they should not be considered inherently minimally invasive. While some approaches genuinely reduce overall surgical burden and postoperative morbidity, others achieve augmentation avoidance through technically demanding procedures that preserve native anatomy without necessarily reducing procedural complexity [17]. Accordingly, augmentation avoidance should be regarded as the overarching treatment philosophy, whereas minimally invasive surgery represents one possible means of achieving this objective in appropriately selected clinical situations [20].

Among the recent developments in implant design, convergent (hyperbolic) transmucosal-neck implants have attracted increasing attention as a minimally invasive alternative aimed at preserving peri-implant hard and soft tissue stability. By positioning the implant–abutment transition above the crestal bone and promoting favorable soft tissue adaptation, this design may reduce biological stress at the transmucosal interface while allowing simplified surgical and prosthetic protocols in appropriately selected cases. Medium- and long-term clinical studies, together with a recent systematic review, have reported favorable outcomes in terms of marginal bone stability, peri-implant soft tissue health, and implant survival [27].

Short and ultra-short implants, narrow-diameter implants, tilted implant configurations, pterygoid and zygomatic implants, immediate loading protocols, flapless surgical approaches, and computer-guided implant placement represent some of the most relevant contemporary augmentation-avoidance strategies in implant dentistry. Although these approaches share the objective of reducing the need for extensive bone augmentation, they differ substantially in terms of surgical invasiveness, technical complexity, and clinical indications [28]. Importantly, minimally invasive rehabilitation in geriatric patients should not be interpreted solely as the pursuit of fixed implant-supported restorations. Implant-retained overdentures continue to represent an important therapeutic option for many elderly and frail individuals, providing substantial improvements in retention, function, comfort, and quality of life while often requiring less extensive surgical intervention than fixed full-arch rehabilitations [29]. Consequently, minimally invasive implant rehabilitation encompasses a broad spectrum of treatment modalities that can be tailored to the anatomical, medical, functional, and psychosocial characteristics of individual patients.

This evolving philosophy reflects a broader transformation in the objectives of implant therapy [11]. In elderly patients, treatment success should not be assessed exclusively according to implant survival rates or radiographic outcomes [30]. Equally important are patient-centered considerations such as postoperative morbidity, treatment burden, recovery time, maintenance requirements, functional independence, quality of life, and the individual’s perception of treatment benefit [31]. Contemporary geriatric implant dentistry therefore requires a personalized and patient-centered approach that integrates anatomical, medical, functional, and psychosocial factors into shared clinical decision-making.

Despite the growing interest in minimally invasive implant therapy, the available literature remains largely fragmented, with most studies and reviews focusing on individual treatment modalities rather than on the broader clinical objective of reducing or avoiding extensive bone augmentation procedures in older adults [32]. Reviews addressing short implants, immediate loading, implant-retained overdentures, tilted implants, or alternative implant placement strategies are available; however, these approaches are frequently discussed separately and within different clinical contexts [33,34]. Furthermore, clinicians are often required to choose among multiple augmentation-avoidance strategies according to patient-specific anatomical, medical, and functional factors, yet practical guidance integrating these therapeutic options within a comprehensive geriatric framework remains limited. To the best of the authors’ knowledge, no comprehensive narrative review has specifically examined the full spectrum of contemporary augmentation-avoidance strategies within the context of geriatric implant rehabilitation.

Therefore, the aim of this comprehensive narrative review is to critically analyze contemporary minimally invasive implant rehabilitation strategies for patients with geriatric characteristics, with particular emphasis on treatment approaches designed to reduce or avoid extensive bone augmentation procedures. Special attention will be given to the biological rationale, clinical indications, advantages, limitations, and patient-centered outcomes associated with these strategies, with the objective of providing a clinically relevant framework for treatment planning and decision-making in contemporary geriatric implant dentistry.

## 2. Materials and Methods

### 2.1. Literature Search Strategy

This comprehensive narrative review was undertaken to provide a clinically oriented and critical synthesis of the contemporary literature concerning minimally invasive implant rehabilitation strategies for geriatric patients, with particular emphasis on treatment approaches aimed at reducing or avoiding extensive bone augmentation procedures. Given the diversity of available evidence, including differences in study designs, patient populations, surgical protocols, prosthetic concepts, and outcome measures, a narrative review framework was considered the most appropriate methodological approach to explore both clinical and conceptual aspects of this topic.

To define the scope of the review, the research question was framed using an adapted PICO structure. The population of interest comprised older adults requiring implant-supported rehabilitation, together with broader adult cohorts reporting findings relevant to older individuals. The interventions included contemporary implant and prosthetic approaches intended to reduce or avoid extensive bone augmentation procedures. Where comparative evidence was available, conventional implant placement associated with bone augmentation, sinus floor elevation, or ridge reconstruction represented the comparator. The outcomes considered included implant and prosthetic survival, marginal bone level changes, biological, surgical, technical, and prosthetic complications, treatment duration, postoperative morbidity, maintenance requirements, oral function, treatment satisfaction, and oral health-related quality of life. Given the heterogeneity of the available evidence and the inclusion of non-comparative studies, this framework was used to clarify the review question rather than as a restrictive eligibility model.

A structured literature search was conducted in PubMed/MEDLINE, Scopus, and Web of Science from database inception to 30 May 2026. The final search update was performed on the same date, and only publications written in English were considered. The objective of the search was not to identify all available evidence according to systematic review methodology, but rather to obtain a comprehensive and clinically meaningful overview of the literature relevant to contemporary minimally invasive implant rehabilitation in older adults.

The search strategy combined Medical Subject Headings (MeSH) and free-text terms related to geriatric implant dentistry, implant rehabilitation, minimally invasive treatment concepts, and bone augmentation avoidance. Search terms were combined using the Boolean operators AND and OR. Truncation and database-specific syntax were applied whenever appropriate. The search strategy was adapted to the indexing systems of each database while preserving the same conceptual domains. The principal search terms included combinations of keywords such as “elderly patients”, “older adults”, “geriatric patients”, “geriatric dentistry”, “dental implants”, “implant rehabilitation”, “short implants”, “ultra-short implants”, “narrow-diameter implants”, “tilted implants”, “All-on-4”, “immediate loading”, “pterygoid implants”, “zygomatic implants”, “trans-sinus implants”, “implant-retained overdentures”, “flapless surgery”, “computer-guided surgery”, “bone augmentation”, “guided bone regeneration”, “convergent-neck implants”, “hyperbolic-neck implants”, “patient-reported outcomes”, “sinus floor elevation”, and “edentulism”.

To improve the comprehensiveness of the review and minimize the possibility of overlooking relevant publications, manual screening of the reference lists of selected articles was performed. Additional hand-searching was conducted in leading journals focusing on implant dentistry, oral surgery, prosthodontics, oral rehabilitation, and geriatric dentistry. Particular attention was given to seminal publications, consensus statements, systematic reviews, meta-analyses, and clinically relevant studies addressing treatment outcomes in elderly and medically compromised populations.

The search strategy was adapted according to the indexing characteristics of each database. A representative search string used for PubMed/MEDLINE is reported below: (“elderly” OR “older adults” OR “geriatric patients”) AND (“dental implants” OR “implant rehabilitation”) AND (“short implants” OR “ultra-short implants” OR “narrow implants” OR “tilted implants” OR “All-on-4” OR “pterygoid implants” OR “zygomatic implants” OR “implant overdentures” OR “immediate loading” OR “guided surgery” OR “flapless surgery”) AND (“bone augmentation” OR “guided bone regeneration” OR “sinus floor elevation” OR “sinus lift”).

Minor adaptations of the search strategy were applied for Scopus and Web of Science according to database-specific indexing systems according to the specific indexing systems and search functionalities of each database.

### 2.2. Literature Selection and Eligibility Considerations

The literature included in this review was selected according to its relevance to the objectives of the study, methodological quality, clinical applicability, and contribution to the understanding of minimally invasive implant rehabilitation strategies in geriatric patients. Given the narrative nature of the review, a broad range of evidence sources was considered appropriate to provide a comprehensive and clinically meaningful overview of the topic.

Eligible publications included randomized clinical trials, prospective and retrospective clinical studies, cohort studies, case–control studies, systematic reviews, meta-analyses, consensus reports, clinical guidelines, and narrative reviews addressing implant rehabilitation approaches that may reduce or avoid extensive bone augmentation procedures. Studies investigating short and ultra-short implants, narrow-diameter implants, tilted implant concepts, pterygoid implants, zygomatic implants, trans-sinus approaches, immediate loading protocols, flapless and computer-guided surgery, implant-retained overdentures, and patient-centered outcomes in elderly populations were considered particularly relevant. Titles and abstracts retrieved through the database searches were screened for their relevance to the objectives of the review. Publications considered potentially eligible subsequently underwent full-text evaluation. The reference lists of the included articles, relevant systematic reviews, and consensus statements were also examined to identify additional publications not retrieved through the electronic database search. Duplicate records were removed prior to the screening process. The literature selection process was performed collaboratively by the authors. Potentially relevant publications were discussed jointly until agreement was reached regarding their inclusion in the narrative synthesis.

Publications were not selected according to predefined quantitative criteria but rather on the basis of their capacity to contribute to a broader understanding of contemporary augmentation-avoidance strategies in geriatric implant dentistry. Preference was given to studies presenting clinically relevant findings, long-term outcomes, patient-centered measures, and evidence that could support treatment planning and clinical decision-making in older adults.

Studies focusing exclusively on laboratory investigations without direct clinical implications, animal studies, isolated technical reports with limited clinical applicability, or topics unrelated to implant rehabilitation in elderly patients were not considered central to the objectives of this review and were therefore excluded from the narrative synthesis.

**Figure 1:**
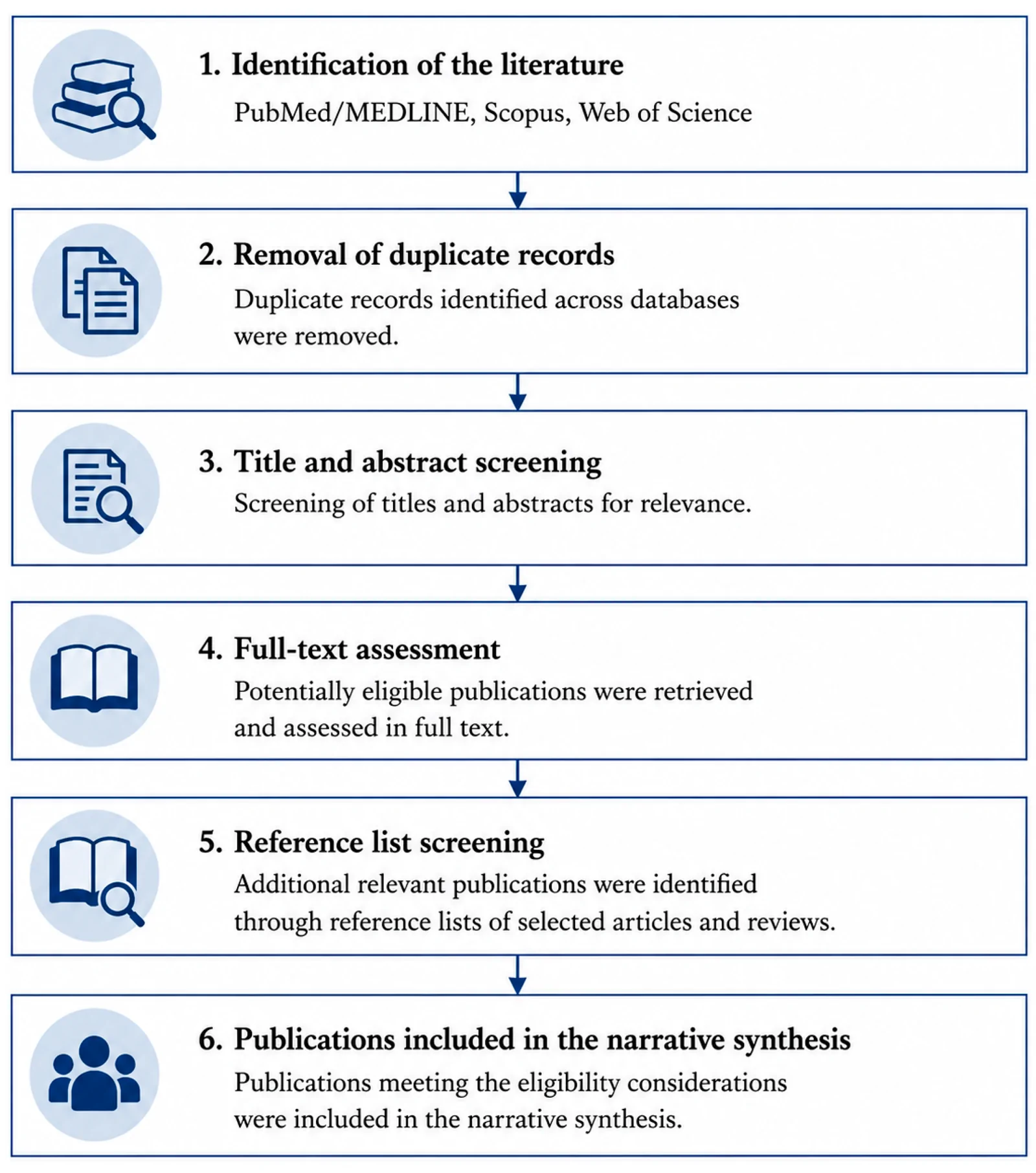
Workflow illustrating the literature identification, screening, selection, and inclusion process adopted for the present narrative review.

The overall workflow adopted for literature identification, screening, and study selection is illustrated in Figure 1.

### 2.3. Data Extraction

Information from the selected publications was extracted using a structured data collection approach developed for the purposes of this narrative review. The information recorded included study design, study objectives, patient population, age characteristics, anatomical condition, implant or rehabilitation strategy, loading and prosthetic protocol, follow-up duration, implant and prosthetic survival, marginal bone level changes, biological, surgical, technical, and prosthetic complications, maintenance requirements, and patient-reported or functional outcomes whenever available. Attention was given to variables specifically relevant to geriatric patients, including frailty, multimorbidity, medication burden, functional dependence, oral hygiene capability, caregiver involvement, and overall treatment burden.

Because this review incorporated a broad spectrum of evidence, including primary clinical studies, systematic reviews, meta-analyses, consensus statements, and narrative publications, not all variables were consistently reported across the available literature. Consequently, the extracted information varied according to the design and objectives of each study, and only the variables relevant to the aims of the present review were considered during data collection.

### 2.4. Evidence Synthesis

Given the substantial heterogeneity of the available literature with respect to study design, patient populations, surgical protocols, implant systems, prosthetic approaches, follow-up duration, and reported outcome measures, a quantitative synthesis was not considered appropriate. Accordingly, the extracted evidence was analyzed qualitatively using a thematic and clinically oriented approach.

The synthesis was organized according to the principal contemporary augmentation-avoidance approaches identified during the literature review, including short and ultra-short implants, narrow-diameter implants, convergent-neck implant designs, tilted implant concepts and full-arch rehabilitation, pterygoid, trans-sinus, and zygomatic implants, immediate loading protocols, flapless and computer-guided surgery, and implant-retained overdentures. Within each thematic section, the available evidence was interpreted in relation to the biological rationale, clinical indications, reported outcomes, potential complications, and considerations specifically relevant to older adults. This thematic organization provided a structured overview of the available evidence across the principal augmentation-avoidance rehabilitation strategies, facilitating comparison of their clinical indications, reported outcomes, and relevance for geriatric patients.

The objective of the synthesis was not to identify a single preferred treatment strategy but rather to recognize recurring patterns, areas of consensus, current controversies, and knowledge gaps that may support clinical decision-making in geriatric implant rehabilitation. Attention was given to factors influencing treatment selection, preservation of native bone, surgical invasiveness, long-term maintenance, functional rehabilitation, patient satisfaction, oral health-related quality of life, and overall treatment burden.

Owing the narrative nature of the review and the diversity of the available evidence, no formal risk-of-bias assessment was performed. Nevertheless, the methodological quality, clinical relevance, consistency of findings, and applicability of the available evidence to contemporary geriatric implant dentistry were critically considered throughout the synthesis and interpretation of the literature.

## 3. Results

The literature search identified a broad range of surgical and prosthetic strategies aimed at reducing treatment invasiveness and minimizing the need for extensive bone augmentation procedures in geriatric patients [35]. The available evidence highlights the influence of anatomical, systemic, and patient-related factors on treatment selection and rehabilitation outcomes. The principal findings are presented according to the clinical challenges reported in elderly populations and the contemporary augmentation-avoidance strategies described in the literature.

### 3.1. Clinical Challenges Driving Minimally Invasive Implant Rehabilitation in Geriatric Patients

The available literature indicates that implant rehabilitation in geriatric patients is frequently influenced by a combination of anatomical, biological, systemic, functional, and patient-related factors [35]. These characteristics are commonly described in studies involving elderly populations and are often considered when evaluating treatment feasibility, rehabilitation complexity, and long-term maintenance requirements (Table 1).

One of the most frequently reported findings in older adults seeking implant-supported rehabilitation is alveolar ridge atrophy [36]. Following tooth loss, progressive bone resorption may result in substantial reductions in ridge width and height, particularly in individuals with prolonged periods of edentulism [37]. Numerous investigations have described varying degrees of horizontal and vertical bone loss in both the maxilla and mandible, with advanced atrophy representing a common feature among patients referred for implant treatment [38,39]. The posterior maxilla and posterior mandible are consistently identified as anatomical regions where reduced bone availability may complicate conventional implant placement [40].

In addition to anatomical changes associated with edentulism, age-related modifications in bone physiology have been documented. Although significant variability exists among individuals, studies have reported changes in bone remodeling activity, vascularization, and regenerative potential that may contribute to differences in bone quantity and quality observed in elderly populations [41]. These biological characteristics are frequently described in investigations evaluating implant treatment in older adults and constitute an important component of the overall clinical picture [30].

Systemic health conditions are likewise well represented in the literature. Epidemiological studies indicate that multimorbidity becomes increasingly prevalent with advancing age, and elderly patients seeking implant rehabilitation frequently present with one or more chronic medical conditions [42]. Cardiovascular diseases, diabetes mellitus, osteoporosis, and neurodegenerative disorders are among the most reported systemic conditions in studies involving implant-supported rehabilitation [43]. In parallel, polypharmacy is frequently encountered in geriatric populations and is regularly described as a relevant characteristic during patient assessment and treatment planning [44].

Over the last decade, increasing attention has been directed toward the concept of frailty. Several investigations have identified frailty as a relevant parameter when evaluating older adults undergoing dental and implant-related interventions [16,45]. Frailty is generally characterized by reduced physiological reserve and increased vulnerability to adverse health outcomes and has become progressively incorporated into contemporary geriatric assessment models [46]. Consequently, frailty is increasingly reported alongside traditional demographic and medical variables in studies involving implant rehabilitation in elderly individuals.

Functional and cognitive characteristics constitute another recurrent topic within the available literature. Reduced manual dexterity, visual impairment, cognitive decline, and varying degrees of dependency are commonly described among older adults receiving implant-supported rehabilitation [34]. These variables are frequently included in studies assessing treatment outcomes and long-term maintenance, reflecting their relevance within geriatric populations [17,47]. Maintenance-related factors have also received increasing attention in recent years. Several investigations have evaluated the influence of oral hygiene capability, attendance at supportive care programs, and long-term compliance on the maintenance of implant-supported rehabilitation [48]. The ability to perform daily oral hygiene procedures and participate in regular follow-up programs is frequently reported when assessing outcomes in elderly populations, particularly among individuals presenting with functional or cognitive limitations [49].

In addition to conventional clinical parameters, contemporary studies increasingly incorporate patient-reported outcome measures into the evaluation of implant rehabilitation. Oral health-related quality of life, masticatory performance, treatment satisfaction, perceived treatment burden, and functional independence are among the outcomes most frequently investigated [50]. These measures are now commonly included alongside traditional indicators such as implant survival and prosthetic success [51].

**Figure 2:**
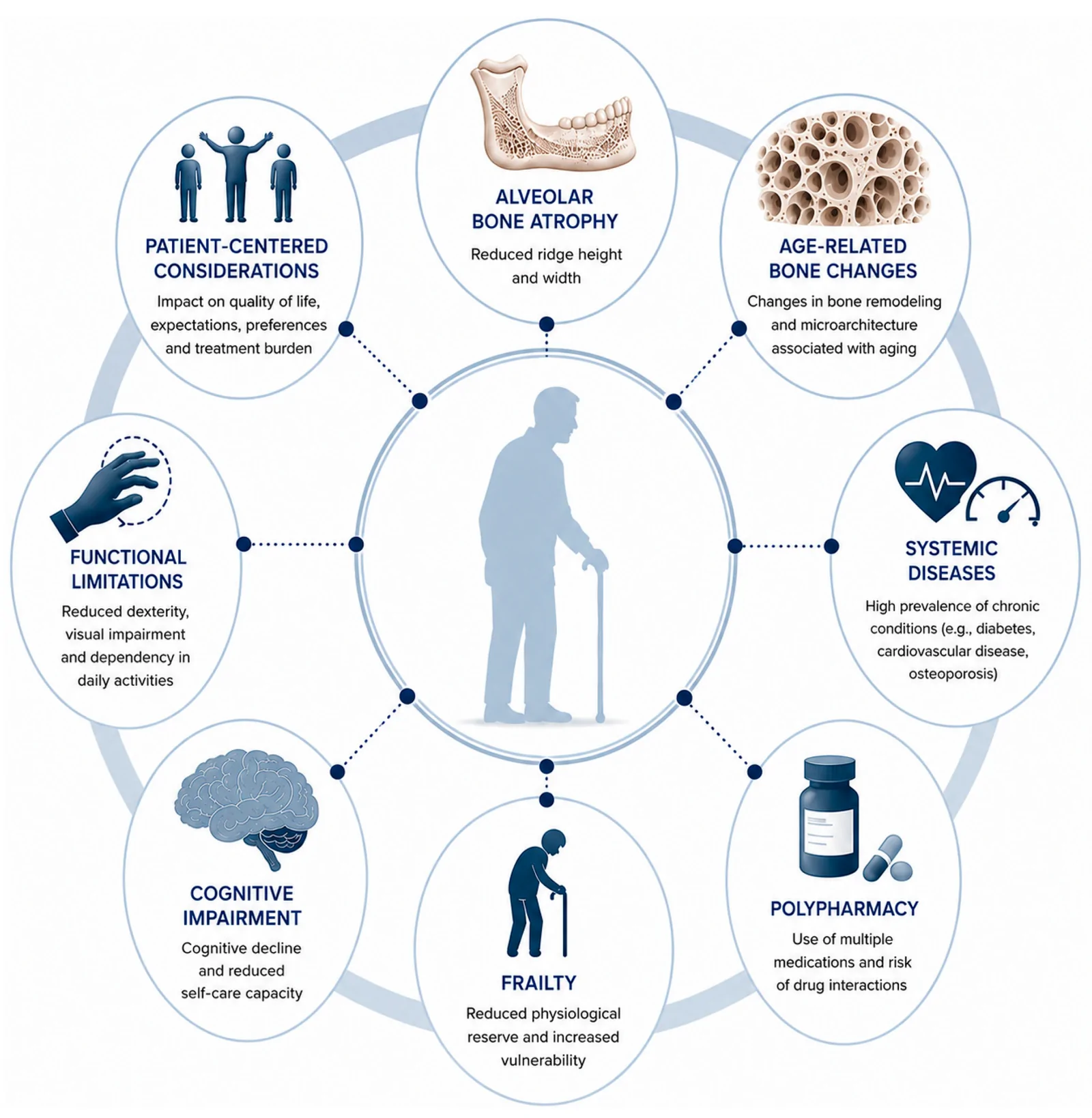
Overview of the principal factors influencing implant rehabilitation planning in older adults.

The principal anatomical, biological, systemic, functional, and patient-related factors reported in the literature are summarized in Table 1 and schematically represented in Figure 2.

### 3.2. Short and Ultra-Short Implants

Short implants have been extensively investigated over the last two decades as a treatment option for rehabilitating edentulous areas characterized by limited vertical bone availability [52]. Although the definition of a short implant has evolved over time, early studies commonly considered implants measuring 10 mm or less as short implants. More recent classifications generally define short implants as implants with an intrabony length of 8 mm or less, whereas ultra-short or extra-short implants are typically described as implants measuring 6 mm or less in length [53].

Current evidence derives from a broad spectrum of clinical investigations, including randomized trials, observational studies, and evidence syntheses, evaluating different implant systems, loading protocols, and prosthetic configurations. Most studies have focused on partially or completely edentulous patients with reduced vertical bone height, particularly in the posterior maxilla and posterior mandible [54,55]. Most clinical investigations have evaluated short implants in anatomically challenging posterior regions where limited vertical bone availability restricts the placement of standard-length implants. The most frequently investigated indications include the posterior maxilla with reduced residual bone height beneath the maxillary sinus and the posterior mandible affected by alveolar resorption and proximity to the inferior alveolar nerve [56,57,58,59].

Implant survival, implant success, and marginal bone stability represent the principal outcomes reported across the available literature, together with biological and prosthetic complications evaluated over short-, medium-, and long-term follow-up periods [60,61,62,63]. Comparative investigations represent another important component of the available evidence. In the posterior maxilla, short implants have frequently been compared with longer implants placed in conjunction with sinus floor elevation procedures [64]. In the posterior mandible, studies have commonly evaluated short implants against longer implants inserted after vertical ridge augmentation procedures [65]. These investigations have generally reported outcomes including implant survival, implant success, marginal bone level changes, biological complications, prosthetic complications, treatment duration, and patient-reported measures.

Interest in ultra-short implants has increased considerably in recent years. Clinical investigations have evaluated implants measuring ≤6 mm, including 5-, 4-, and, less frequently, 3 mm designs, in both fixed and removable rehabilitations ranging from single crowns to full-arch prostheses and implant-retained overdentures [66,67,68,69]. As with short implants, the literature primarily reports implant survival, marginal bone stability, and biological and prosthetic complications, while also examining factors such as crown-to-implant ratio, prosthetic design, splinting protocols, implant location, loading conditions, and medium- to long-term clinical outcomes [70,71].

Patient-centered outcomes have received increasing attention in studies evaluating both short and ultra-short implants. Oral health-related quality of life, treatment satisfaction, masticatory performance, and perceived treatment burden are among the most frequently reported patient-centered outcomes [72]. However, these measures remain less consistently reported than traditional clinical and radiographic parameters.

Evidence specifically focused on geriatric patients remains limited, as most studies include mixed adult cohorts rather than populations specifically designed to investigate older adults or frail individuals [30]. Consequently, current knowledge is largely extrapolated from heterogeneous adult populations, although older adults are frequently represented within prospective and retrospective clinical studies evaluating short and ultra-short implant rehabilitation.

### 3.3. Narrow-Diameter Implants

Narrow-diameter implants (NDIs) have been extensively investigated as a treatment option in clinical situations characterized by limited horizontal bone availability or reduced mesiodistal space [73]. Although different classifications have been proposed, NDIs are generally defined as implants with diameters smaller than conventional implant dimensions [74]. One of the most frequently cited classifications distinguishes Category 1 implants (<3.0 mm), Category 2 implants (3.0–3.25 mm), and Category 3 implants (3.3–3.5 mm). Some classifications additionally include mini-implants, which are generally described as implants with diameters below 3.0 mm [75].

Current evidence on NDIs derives from a broad spectrum of clinical investigations and evidence syntheses evaluating different implant systems, loading protocols, and prosthetic configurations in both partially and completely edentulous patients. These studies encompass anterior and posterior regions of the maxilla and mandible and include both fixed and removable implant-supported rehabilitations. Most clinical investigations have focused on anatomically narrow alveolar ridges or reduced mesiodistal spaces that limit the placement of conventional-diameter implants [76,77]. Frequently reported indications include replacement of maxillary lateral incisors, mandibular incisors, and other teeth located in areas with limited prosthetic space, although NDIs have also been investigated in posterior regions with reduced ridge width [78,79].

The evidence base includes studies evaluating NDIs supporting single crowns, fixed partial prostheses, full-arch rehabilitations, and removable prosthetic restorations. A considerable number of investigations have focused on mandibular overdenture treatment concepts, where narrow-diameter implants have been evaluated under different loading protocols and follow-up durations [80]. Both splinted and unsplinted treatment approaches have been described, and studies have assessed a variety of attachment systems and prosthetic designs [81].

Implant survival, implant success, and marginal bone stability represent the principal outcomes reported for NDIs across short-, medium-, and long-term follow-up periods in both fixed and removable rehabilitations [82,83]. Biological and prosthetic complications have been extensively investigated in both fixed restorations and implant-retained overdentures [84,85]. Attention has also been devoted to the mechanical performance of NDIs, with studies evaluating implant and abutment fracture, prosthetic screw loosening, component failure, and other technical complications in relation to implant diameter, prosthetic design, implant location, loading conditions, and follow-up duration [86]. Comparative studies have evaluated NDIs against conventional-diameter implants while also investigating the influence of implant diameter, splinting protocols, prosthetic configuration, and loading conditions on clinical outcomes. Reported endpoints include implant survival, marginal bone stability, biological, prosthetic, and mechanical complications, together with patient-reported outcomes [83,87,88,89].

Patient-reported outcome measures have been increasingly incorporated into studies evaluating NDIs. Oral health-related quality of life, treatment satisfaction, prosthesis stability, masticatory function, and patient perception of treatment have been assessed in several clinical investigations [90]. However, as observed for other implant treatment modalities, patient-reported outcomes remain less consistently reported than traditional clinical and radiographic parameters.

### 3.4. Tilted Implants and Full-Arch Concepts

Tilted implants and full-arch rehabilitation concepts have been extensively investigated for the rehabilitation of edentulous patients. Current evidence derives from a broad spectrum of clinical investigations and evidence syntheses evaluating different combinations of axial and tilted implants placed in the maxilla and mandible across various clinical settings and follow-up periods [91,92,93,94].

**Figure 3:**
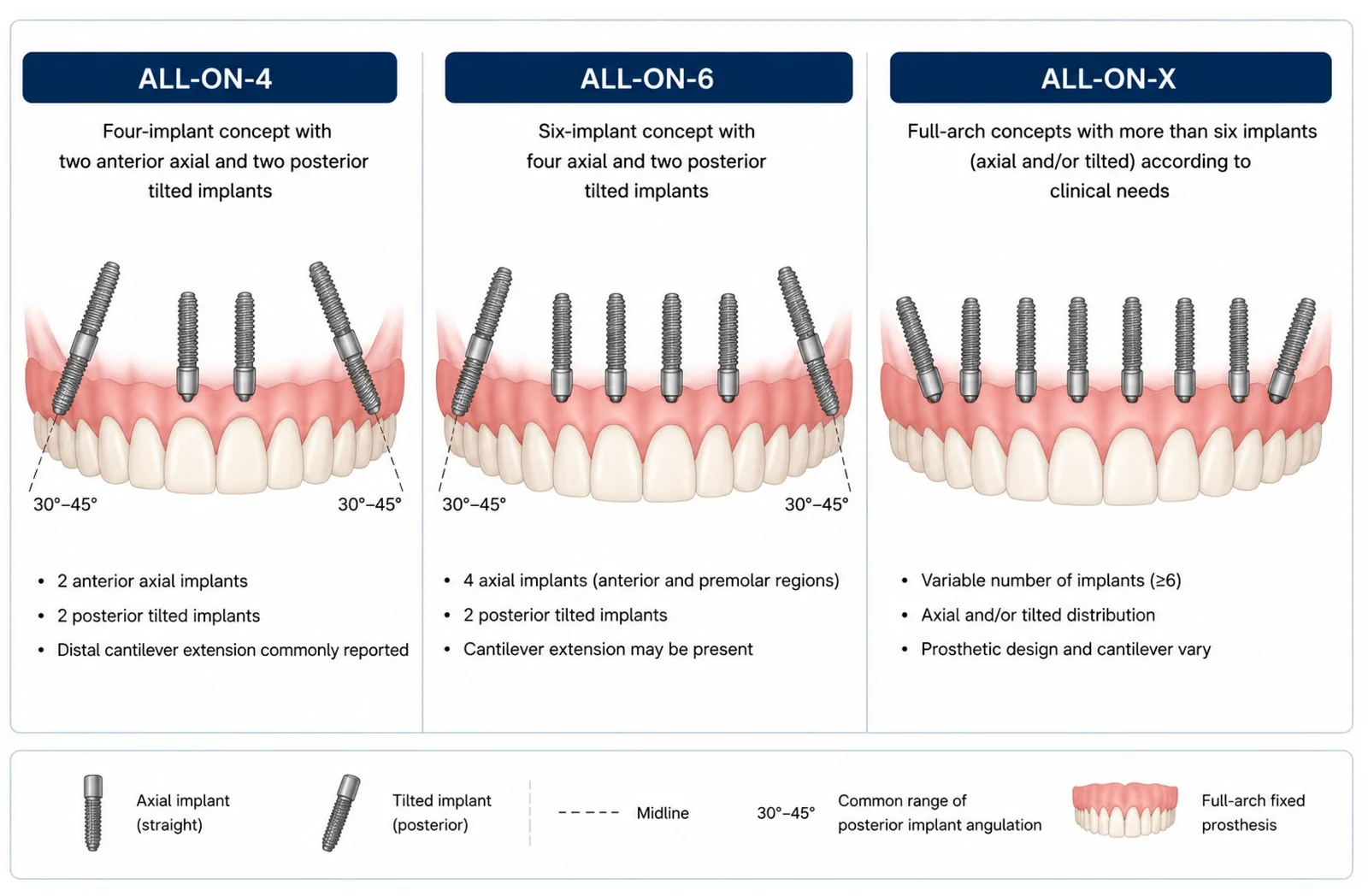
**Schematic illustration of the most commonly described full-arch implant rehabilitation concepts, including All-on-4, All-on-6, and other full-arch configurations employing combinations of axial and tilted implants.** The figure is intended for illustrative purposes only. Implant number, distribution, angulation, cantilever extension, and prosthetic design should be individualized according to patient-specific anatomical conditions, biomechanical requirements, and prosthetic treatment planning and should not be interpreted as standardized or interchangeable treatment protocols.

Several full-arch configurations have been described in the literature. The most frequently reported treatment concepts include All-on-4, All-on-6, and other full-arch protocols employing combinations of axial and tilted implants [95,96,97]. Clinical investigations have evaluated treatment approaches involving anterior axial implants combined with posterior tilted implants, as well as configurations incorporating a greater number of implants distributed throughout the arch [98]. These concepts have been described for both maxillary and mandibular rehabilitations across different degrees of alveolar ridge resorption [91,92]. Figure 3 schematically illustrates the most reported full-arch rehabilitation concepts. As discussed above, these configurations represent conceptual examples rather than standardized treatment protocols, and implant number, distribution, angulation, and prosthetic design should always be individualized according to anatomical and biomechanical requirements.

Most published studies involve completely edentulous patients rehabilitated with fixed implant-supported prostheses, evaluating different implant systems, surgical techniques, prosthetic materials, and restorative designs. Both immediate provisionalization protocols and definitive full-arch rehabilitations have been extensively investigated [99,100].

Implant survival, prosthetic survival, and marginal bone stability represent the principal outcomes reported across short-, medium-, and long-term follow-up periods, with several cohort studies demonstrating favorable outcomes beyond five years of observation [101,102,103]. Biological complications have also been extensively investigated and include implant loss, peri-implant mucositis, peri-implantitis, and other biological events occurring during follow-up [104]. Prosthetic complications have likewise been frequently reported and encompass a broad spectrum of technical events affecting implant-supported full-arch rehabilitations [105]. Framework complications, prosthetic fractures, veneer fractures, attachment-related complications, and prosthetic screw-related events are among the outcomes commonly described in the literature [106].

Attention has been devoted to implant angulation, anterior–posterior spread, cantilever extension, implant distribution, and framework design because of their potential influence on biomechanical behavior and long-term clinical performance [107,108]. Immediate loading represents one of the most extensively investigated components of full-arch rehabilitation [109]. Clinical studies have compared immediate, early, and conventional loading protocols while evaluating clinical, prosthetic, radiographic, maintenance-related, and patient-centered outcomes across different implant configurations [110]. Long-term maintenance requirements, including prosthetic interventions, replacement of restorative components, management of technical complications, supportive care, oral hygiene, and peri-implant tissue health, have also received increasing attention [111,112].

Evidence specifically focused on geriatric patients remains limited. Nevertheless, because complete edentulism is more prevalent among older adults, many full-arch rehabilitation studies include patients aged 65 years and older. Consequently, current knowledge is largely derived from mixed adult cohorts in which clinical, prosthetic, maintenance-related, and patient-centered outcomes in older adults have been reported, rather than from studies specifically designed for frail geriatric populations.

### 3.5. Pterygoid and Trans-Sinus Implants

Pterygoid and trans-sinus implants have been investigated as augmentation-avoidance strategies for rehabilitation of severely atrophic posterior maxillae with limited residual bone availability [113,114]. Most published studies concerned edentulous or severely resorbed maxillae requiring implant-supported rehabilitation without extensive sinus augmentation procedures [115,116].

Pterygoid implants have been evaluated in patients presenting severe posterior maxillary resorption, where implant anchorage is extended toward the pterygoid region through the posterior maxilla [117]. Their use has been reported in both partially and completely edentulous individuals, although a substantial proportion of the available evidence concerns fixed full-arch rehabilitations [118,119]. The literature has assessed different implant lengths, implant angulations, loading protocols, and prosthetic configurations. Pterygoid implants are frequently described in conjunction with anterior axial implants and, in some treatment concepts, with tilted posterior implants distributed throughout the arch [120].

Trans-sinus implants represent another treatment approach described in the literature for the rehabilitation of the atrophic posterior maxilla [121]. Their application has been investigated in situations characterized by limited residual bone height beneath the maxillary sinus and advanced alveolar ridge resorption [113]. Published reports have described trans-sinus implants supporting fixed implant-supported prostheses in both partially and completely edentulous patients [121]. Different implant trajectories, implant designs, prosthetic configurations, and loading protocols have been documented across the available literature. In many studies, trans-sinus implants have been incorporated into treatment concepts involving axial and tilted implants within the same prosthetic rehabilitation [122,123].

A substantial proportion of the available evidence concerns full-arch rehabilitation concepts. Reports have described pterygoid and trans-sinus implants in completely edentulous maxillae rehabilitated with fixed implant-supported prostheses [124]. Treatment protocols have included various combinations of axial, tilted, pterygoid, and trans-sinus implants distributed throughout the arch. Different framework designs, cantilever extensions, and prosthetic support configurations have also been reported within full-arch rehabilitations [125].

Implant survival, prosthetic survival, marginal bone stability, and biological complications represent the principal outcomes reported across short-, medium-, and long-term follow-up periods [115,126,127]. Frequently described biological events include implant loss, peri-implant mucositis, and peri-implantitis. In addition to implant- and prosthesis-related outcomes, several investigations have evaluated surgical complications, postoperative events, and sinus-related morbidity, particularly for trans-sinus rehabilitation protocols [115,128]. Evidence specifically focused on geriatric patients remains limited. Although older adults are frequently represented in studies of rehabilitation of the atrophic maxilla, current knowledge is largely derived from mixed adult populations rather than dedicated geriatric cohorts.

### 3.6. Zygomatic Implants

Zygomatic implants have been extensively investigated in the rehabilitation of severely and extremely atrophic maxillae characterized by advanced bone resorption and limited residual bone availability [129]. Most published reports concern individuals presenting long-standing edentulism, advanced maxillary atrophy, severe posterior maxillary bone loss, or previous treatment histories associated with extensive maxillary resorption [130,131].

The literature describes a wide variety of rehabilitation concepts, including unilateral and bilateral zygomatic implants, hybrid rehabilitations combining zygomatic implants with conventional, tilted, or pterygoid implants, and quad zygoma protocols [132]. These approaches have been evaluated within fixed full-arch rehabilitations using different implant distributions, framework designs, restorative materials, and prosthetic protocols, including both provisional and definitive restorations [130,133,134].

Immediate-function protocols are commonly reported within the available literature. Numerous investigations have evaluated immediate loading following placement of zygomatic implants, while others have reported outcomes associated with early or conventional loading approaches [135]. Reported outcome measures include implant survival, prosthetic survival, peri-implant tissue conditions, radiographic findings, and indicators of long-term implant performance [136]. Follow-up periods vary considerably among studies and include both short-term and long-term observations. Marginal bone level changes, soft tissue health, and other clinical and radiographic parameters have been assessed at different observation intervals [137].

Surgical outcomes constitute a major focus of the available literature and include intraoperative events, postoperative complications, sinus-related morbidity, and soft tissue complications associated with different surgical protocols [138]. Prosthetic maintenance has also been widely investigated, with studies reporting framework fractures, prosthetic screw-related events, component replacement, and other technical interventions required during long-term follow-up [139]. Patient-reported outcome measures have received increasing attention in studies evaluating zygomatic implant-supported rehabilitation [140]. Treatment satisfaction, oral health-related quality of life, masticatory function, speech-related outcomes, comfort, and patient-perceived esthetic results are among the variables most frequently assessed [140]. Evidence specifically focused on geriatric populations remains limited. Nevertheless, many clinical studies involve patients presenting conditions frequently associated with advanced age, including long-term edentulism, severe maxillary atrophy, and extensive maxillary bone loss. Older adults are represented within numerous cohorts evaluating zygomatic implant-supported rehabilitation, particularly in studies involving fixed complete-arch prostheses. However, dedicated investigations specifically designed to evaluate outcomes in geriatric populations remain relatively scarce.

### 3.7. Immediate Loading Protocols

The timing of prosthetic loading represents one of the most extensively investigated variables in implant dentistry [141]. Immediate loading, immediate restoration, and immediate-function protocols have been evaluated across a broad spectrum of implant-supported rehabilitations within contemporary implant rehabilitation. Clinical investigations have examined immediate loading in single-tooth restorations, partial rehabilitations, complete-arch prostheses, and implant-retained overdentures [142]. These protocols have been reported in both maxillary and mandibular arches and have been assessed across different surgical and prosthetic treatment concepts. Immediate loading has also been investigated in conjunction with a variety of implant configurations and rehabilitation strategies described throughout the implant literature [143].

A recurrent focus of the available evidence concerns factors associated with implant stability at the time of loading. Studies have reported insertion torque values, primary stability measurements, resonance frequency analysis, and implant stability assessments as variables frequently evaluated in relation to immediate-function protocols [144]. Different methods for assessing implant stability have been described, and these parameters have often been reported alongside clinical and radiographic outcomes during follow-up [145]. Comparative investigations commonly evaluate immediate loading in relation to early and conventional loading protocols [146]. These studies have assessed treatment outcomes across different observation periods and clinical settings, examining loading strategies in relation to implant location, prosthetic design, implant distribution, and rehabilitation type. Immediate restoration and immediate-function approaches have likewise been investigated within a variety of prosthetic treatment protocols [147].

Patient-centered outcomes constitute an important component of the literature on immediate loading protocols [148]. Frequently assessed variables include treatment satisfaction, oral health-related quality of life, masticatory function, comfort, speech, and patient-perceived treatment experience [149]. Immediate loading has been evaluated in both fixed and removable rehabilitations, including implant-retained overdentures, complete-arch prostheses, and partial restorations using different attachment systems, restorative materials, framework designs, and prosthetic configurations [18,150].

Older adults are well represented in studies evaluating immediate loading protocols, particularly those involving complete edentulism and full-arch implant rehabilitation. Nevertheless, evidence specifically focused on geriatric patients remains limited, and current knowledge is largely extrapolated from mixed adult cohorts rather than from studies specifically designed for frail older individuals.

### 3.8. Flapless and Computer-Guided Surgery

Flapless and computer-guided surgical approaches have become increasingly established in contemporary implant dentistry and have been evaluated across a broad spectrum of rehabilitation protocols in partially and completely edentulous patients [151]. Current evidence derives from clinical investigations and evidence syntheses assessing different surgical workflows in single-tooth restorations, partial rehabilitations, complete-arch prostheses, and implant-retained overdentures [152,153].

Flapless implant surgery has been investigated in both maxillary and mandibular arches using conventional and immediate implant placement, immediate-function protocols, and complete-arch rehabilitations [154,155]. Reported outcomes include clinical and radiographic performance, peri-implant tissue health, surgical parameters, and different approaches to implant site preparation, soft tissue management, and prosthetic rehabilitation [156,157].

Computer-guided implant surgery has likewise been extensively investigated in relation to prosthetically driven treatment planning and implant placement [158]. Both static guided surgery and dynamic navigation systems have been evaluated in a wide range of rehabilitation protocols [159,160]. Studies have reported the use of these approaches in partially and completely edentulous patients, as well as in treatment concepts involving immediate loading and full-arch implant-supported prostheses [161]. Different planning strategies, guide designs, and navigation protocols have been reported across the available evidence utilizing artificial intelligence, which is increasingly being used in the field of dental implantology [162].

One of the principal areas of investigation concerns surgical accuracy and the correspondence between planned and achieved implant positions [163]. Frequently reported outcomes include coronal, apical, angular, and depth deviations evaluated across different implant locations, guide designs, navigation systems, and rehabilitation concepts, with several reviews also comparing alternative guidance workflows [164,165,166].

Reported clinical outcomes include implant and prosthetic survival, marginal bone stability, peri-implant tissue health, biological and technical complications, and prosthetic maintenance requirements evaluated over different follow-up periods [167,168]. Surgical variables such as treatment duration, intraoperative events, and postoperative recovery have also been investigated [169]. In parallel, increasing attention has been devoted to patient-centered outcomes, including treatment satisfaction, oral health-related quality of life, comfort, esthetics, functional performance, and overall treatment experience [170,171]. Older adults are well represented in studies evaluating flapless and computer-guided implant surgery, particularly in complete edentulism, full-arch rehabilitation, and complex prosthetic treatments [18,172]. Nevertheless, evidence specifically focused on geriatric patients remains limited, and age-specific outcomes are rarely reported separately, with current knowledge largely derived from mixed adult populations.

### 3.9. Implant-Retained Overdentures

Implant-retained overdentures represent one of the most extensively investigated treatment options for the rehabilitation of edentulous patients and are frequently reported within the geriatric dentistry literature [173]. Research in this field encompasses prospective and retrospective clinical studies, randomized controlled trials, systematic reviews, umbrella reviews, and meta-analyses evaluating a wide range of prosthetic designs, implant configurations, attachment systems, and treatment protocols in older and adult edentulous populations [174,175].

Mandibular implant-retained overdentures constitute the most frequently investigated rehabilitation concept, particularly among completely edentulous older adults [176]. Maxillary overdentures have also been widely evaluated, although the available literature is less extensive than those concerning mandibular rehabilitations [177]. Published studies include assessments of single-implant, two-implant, and multiple-implant overdenture designs, as well as different implant distributions and support configurations [178]. Both conventional and immediate loading protocols have been reported, together with a variety of prosthetic rehabilitation strategies for completely edentulous patients.

A broad range of attachment systems has been described throughout the available evidence. Ball attachments, locator attachments, bar-retained overdentures, magnetic attachments, and other retention mechanisms have been evaluated under different clinical conditions [179]. Comparative investigations have assessed outcomes associated with alternative attachment systems, maintenance requirements, prosthetic complications, attachment wear, and long-term prosthetic performance across varying follow-up periods [180]. Biological complications, technical complications, prosthetic maintenance requirements, and attachment-related events have likewise been assessed in short-, medium-, and long-term investigations [181].

Patient-reported outcome measures represent a major component of the literature on implant-retained overdentures [29]. Treatment satisfaction, oral health-related quality of life, patient preference, comfort, perceived stability, and overall treatment experience are among the variables most frequently assessed [29]. These outcomes have been evaluated using a variety of validated instruments and are commonly reported alongside conventional clinical and radiographic parameters.

Functional outcomes have received considerable attention across the available literature. Investigations have evaluated masticatory performance, chewing efficiency, oral function, speech-related outcomes, and patient-reported functional measures in edentulous individuals rehabilitated with implant-retained overdentures [182]. Assessments of dietary habits and nutrition-related variables have also been reported in some studies, although less consistently than clinical, prosthetic, and patient-reported outcomes [183].

A substantial proportion of the available evidence specifically involves older adults. Many investigations evaluating implant-retained overdentures have been conducted in elderly populations, including community-dwelling older individuals, medically compromised patients, and residents of long-term care facilities [175]. Age-related factors, oral functional limitations, edentulism-related disability, and prosthetic rehabilitation needs are frequently reported characteristics of the study populations included in this literature [184]. The literature also includes studies examining implant-retained overdentures in relation to frailty, functional dependence, and age-associated changes affecting oral health and prosthetic rehabilitation [30,175]. Clinical, functional, prosthetic, and patient-reported outcomes have been evaluated across diverse geriatric populations, and age-specific variables have been incorporated into a growing number of investigations involving older edentulous patients.

Table 2 summarizes the principal augmentation-avoidance rehabilitation strategies identified in the reviewed literature and integrates their clinical applications, reported outcomes, potential considerations in older adults, and key decision-making considerations that may support individualized treatment planning.

## 4. Discussion

Over the past two decades, implant dentistry has witnessed a progressive shift from a predominantly reconstructive philosophy toward treatment approaches that increasingly seek to exploit existing anatomical structures [24]. This evolution has been driven by the development of alternative implant and prosthetic strategies capable of reducing the need for extensive bone augmentation procedures in selected clinical situations. The literature reviewed in the present study reflects this transition and highlights a growing tendency to adapt treatment planning to the available anatomy rather than routinely modifying anatomy to accommodate implant placement [185].

This paradigm is particularly relevant in geriatric patients. Although advanced age itself does not preclude implant therapy, older adults frequently present clinical characteristics that may influence treatment selection and long-term outcomes [17,30]. Multimorbidity, polypharmacy, frailty, reduced functional reserve, cognitive decline, and limitations in self-care often coexist with anatomical changes associated with long-term edentulism [9]. This clinical complexity also reflects the close relationship between oral health and non-communicable chronic diseases in older adults. Shared behavioral and social determinants contribute to both oral and systemic disease burden, while conditions such as diabetes mellitus, cardiovascular disease, osteoporosis, and neurodegenerative disorders may influence healing capacity, oral function, self-care ability, and the long-term maintenance of implant-supported rehabilitation. These considerations further support the need for an integrated, patient-centered approach in which implant therapy is planned within the broader context of multimorbidity rather than as an isolated oral intervention [186,187]. Consequently, the rationale for avoiding extensive regenerative procedures extends beyond surgical considerations and encompasses the broader objective of reducing overall treatment burden while maintaining functional rehabilitation [9,32]. Within this context, augmentation-avoidance strategies may offer valuable alternatives when treatment complexity, healing time, maintenance requirements, or patient tolerance become relevant factors in clinical decision-making.

An important observation emerging from the reviewed literature is that the available strategies occupy different positions along a continuum of anatomical complexity. Short and ultra-short implants, as well as narrow-diameter implants, are most reported in situations characterized by moderate vertical or horizontal bone deficiencies [188,189]. Tilted implant concepts and full-arch rehabilitation protocols are frequently described in edentulous arches where optimization of residual bone anatomy allows avoidance of additional grafting procedures [91,190]. In contrast, pterygoid, trans-sinus, and zygomatic implants are predominantly investigated in severely atrophic maxillae, where conventional implant placement may be challenging without advanced reconstructive interventions [191]. These observations suggest that the available evidence should not be interpreted as supporting a hierarchical ranking of augmentation-avoidance strategies. Rather, each approach presents distinct biological rationale, surgical demands, maintenance implications, and suitability according to the anatomical and clinical characteristics of the individual patient.

At the same time, the reviewed evidence demonstrates that reducing the need for bone regeneration does not necessarily equate to reducing treatment complexity [192]. A similar distinction should be made between augmentation avoidance and minimally invasive surgery. Although several augmentation-avoidance strategies reduce the need for regenerative procedures, they differ substantially in terms of surgical invasiveness, technical complexity, anatomical risk, and operator experience. Short implants and flapless approaches may genuinely reduce overall surgical burden, whereas pterygoid, trans-sinus, and zygomatic implants primarily represent anatomy-driven alternatives that avoid grafting without necessarily reducing procedural complexity. In many instances, the avoidance of grafting procedures is accompanied by a shift toward alternative surgical, prosthetic, or technical challenges. Short implants and narrow-diameter implants may reduce surgical invasiveness but require careful prosthetic planning and biomechanical management [193,194]. Similarly, pterygoid, trans-sinus, and zygomatic implants may eliminate the need for extensive augmentation while introducing higher levels of surgical complexity and operator dependence [195]. This shift in complexity is a recurring theme across the contemporary literature and highlights the importance of evaluating treatment burden in a comprehensive manner rather than considering bone graft avoidance as an isolated objective. Consequently, treatment selection should be guided not only by implant survival or marginal bone stability, but also by procedural complexity, expected maintenance burden, operator expertise, and the overall health and functional status of the older patient. Similarly, augmentation-avoidance strategies involving pterygoid, trans-sinus, and zygomatic implants should not be regarded solely as alternatives to bone grafting, since they may introduce procedure-specific risks, require advanced surgical expertise, and increase the complexity of long-term prosthetic maintenance, particularly if functional independence or oral hygiene capability deteriorate over time.

Another noteworthy trend concerns the increasing recognition of patient-centered outcomes [196]. Traditional implant dentistry has largely focused on implant survival, prosthetic survival, marginal bone level changes, and radiographic parameters. These outcomes remain fundamental for assessing treatment performance; however, they may not fully capture the dimensions of success that are most relevant to older adults [30]. Functional independence, oral hygiene capability, prosthetic maintainability, comfort, treatment satisfaction, and oral health-related quality of life may have equal or greater clinical relevance in geriatric populations [197]. The growing emphasis on reducing treatment burden is consistent with broader research trends focused on the modulation of inflammation, postoperative recovery, and bone healing in oral rehabilitation [198]. Although patient-reported outcomes have received increasing attention over the last decade, the literature remains largely dominated by implant-centered endpoints. The growing body of evidence evaluating oral function, masticatory performance, quality of life, and patient satisfaction represents an important development; however, these outcomes remain inconsistently reported and are often treated as secondary measures rather than primary endpoints. Interpretation of these findings is further complicated by the considerable heterogeneity of the patient-reported outcome measures (PROMs) adopted across studies [199]. Different assessment instruments, variations in baseline patient characteristics, follow-up intervals, and outcome definitions, together with the potential influence of response bias, limit direct comparisons and make it difficult to determine whether the reported improvements consistently translate into clinically meaningful benefits [200]. Importantly, patient-centered assessment should also include the long-term maintainability of the selected rehabilitation. In geriatric patients, progressive frailty, declining manual dexterity, cognitive impairment, and reduced caregiver support may substantially influence oral hygiene effectiveness, prosthesis maintenance, and the long-term sustainability of implant-supported rehabilitation, regardless of their initial technical success. Furthermore, outcomes particularly relevant to geriatric care, including nutritional status, functional independence, caregiver burden, adaptation to future dependency, and the ability to maintain adequate oral hygiene over time, remain inconsistently evaluated despite their potential influence on long-term rehabilitation success [201].

The literature on implant-retained overdentures illustrates this shift particularly well. Compared with many studies evaluating fixed implant-supported rehabilitation, overdenture research has more frequently incorporated functional and patient-centered measures into outcome assessment [202]. This may explain why overdentures continue to occupy a prominent role within geriatric implant dentistry despite the increasing popularity of fixed solutions [184]. For many older adults, treatment success may be more closely associated with prosthetic stability, ease of hygiene, maintenance feasibility, and functional improvement than with the distinction between fixed and removable prostheses alone. These considerations reinforce the importance of aligning rehabilitation strategies with individual patient priorities and capabilities. Conversely, highly complex fixed rehabilitations may become progressively more difficult to maintain if functional independence declines over time. Consequently, long-term maintenance requirements and the anticipated evolution of patient autonomy should be considered during treatment planning together with anatomical and prosthetic factors.

Immediate loading protocols and digital surgical workflows further reflect the ongoing transformation of implant rehabilitation [28]. The growing adoption of immediate-function concepts demonstrates a shift toward treatment approaches aimed at reducing therapeutic disruption and maintaining prosthetic continuity [202]. Likewise, flapless and computer-guided surgery have expanded the possibilities for prosthetically driven treatment planning and precise implant placement in anatomically challenging situations. Similarly, innovations in implant macrodesign, including convergent transmucosal-neck implants, reflect the same biological philosophy of minimizing surgical trauma while promoting stable peri-implant hard and soft tissue integration over time [27]. None of these rehabilitation strategies should be regarded as universally applicable [152]. Their clinical performance remains strongly influenced by patient selection, anatomical conditions, prosthetic design, primary stability, operator experience, and adherence to appropriate clinical protocols. Rather than supporting a single preferred treatment approach, the available evidence highlights the importance of individualized rehabilitation based on a careful balance between surgical complexity, anatomical conditions, systemic health, frailty, functional expectations, long-term maintenance requirements, and operator expertise. Importantly, the available evidence should not be interpreted as suggesting that augmentation-avoidance strategies universally replace conventional bone augmentation procedures. Rather, these approaches may represent appropriate alternatives only in carefully selected patients when anatomical conditions, systemic health, prosthetic requirements, operator expertise, and long-term maintenance considerations are appropriately balanced.

Despite the substantial volume of published research, several limitations affect the interpretation of the available evidence. Direct comparisons among augmentation-avoidance strategies remain difficult because of considerable heterogeneity in study design, patient populations, implant systems, loading protocols, prosthetic concepts, follow-up duration, and outcome definitions [203]. Additional methodological limitations should also be acknowledged. Many available studies are retrospective in nature, prospective comparative investigations remain limited for several treatment approaches, and definitions of treatment success, complications, and maintenance outcomes are not consistently standardized. Furthermore, patient-reported outcomes are assessed using heterogeneous instruments, which complicates comparisons across studies and limits the ability to perform meaningful synthesis of findings. Moreover, long-term maintenance burden, prosthesis retrievability, caregiver involvement, and changes in functional independence remain inconsistently reported across the available literature despite their particular relevance in geriatric populations.

An additional concern relates to the representation of geriatric populations within the implant literature. Older adults are frequently included in clinical investigations, yet they are often analyzed within broader adult cohorts rather than as a distinct clinical population [30]. Consequently, factors such as frailty, multimorbidity, cognitive impairment, functional dependence, caregiver involvement, oral hygiene capability, and long-term maintenance burden remain insufficiently explored. This limitation is particularly relevant because these variables may substantially influence treatment success and sustainability in routine geriatric care. Future investigations should also adopt more consistent definitions of geriatric populations by integrating chronological age with validated measures of frailty, functional status, and multimorbidity, thereby improving the applicability and comparability of evidence across studies [204].

Beyond its prognostic significance, frailty should also be regarded as a practical component of treatment planning in geriatric implant dentistry. Frailty assessment may assist clinicians in selecting the most appropriate rehabilitation strategy by balancing procedural complexity against the patient’s physiological reserve, functional status, expected treatment burden, and long-term maintenance capacity [205]. In addition to influencing candidacy for implant therapy, frailty may contribute to decisions regarding surgical invasiveness, loading protocols, prosthetic design, maintenance planning, and shared decision-making with patients and caregivers. Although no single assessment tool has been universally adopted in implant dentistry, validated instruments such as the Clinical Frailty Scale (CFS) and the Comprehensive Geriatric Assessment (CGA) may complement conventional anatomical evaluation by providing a broader appraisal of biological age, functional independence, cognition, comorbidity, nutrition, and social support [205,206]. Future research should further investigate the integration of frailty assessment into implant treatment planning to improve individualized care and long-term rehabilitation outcomes in older adults.

The narrative design of the present review should also be considered when interpreting the findings. Unlike systematic reviews and meta-analyses, narrative reviews do not provide quantitative synthesis of the evidence and are inherently influenced by study selection and interpretative judgment. However, the narrative approach allows integration of heterogeneous evidence originating from multiple implant rehabilitation strategies, facilitating a broader understanding of contemporary approaches that seek to reduce the need for extensive bone regeneration in older adults.

Future research should place greater emphasis on variables that are particularly relevant to geriatric populations. Frailty assessment, multimorbidity profiles, medication burden, cognitive status, oral hygiene capability, caregiver involvement, and maintenance requirements should be incorporated more consistently into study design. Greater standardization of patient-reported outcomes and functional measures would also improve comparability across studies. In addition, prospective investigations specifically designed for older adults are needed to clarify how age-related factors influence treatment selection, long-term maintenance, and rehabilitation outcomes. Such evidence would support a transition from technique-centered decision-making toward a more comprehensive geriatric model of implant rehabilitation.

In the end, the available literature supports a progressive transition toward anatomy-driven and patient-centered rehabilitation strategies that may reduce reliance on extensive bone regeneration in selected older adults. Rather than identifying a single optimal treatment pathway, the evidence emphasizes the importance of matching the chosen rehabilitation strategy to the anatomical conditions, functional needs, systemic health status, and long-term maintenance capacity of each individual patient.

## 5. Conclusions

The available literature suggests that contemporary implant rehabilitation offers several strategies that may reduce the need for extensive bone regeneration in appropriately selected patients, although the available evidence remains heterogeneous and geriatric-specific data are still limited. In geriatric patients, treatment planning should extend beyond anatomical considerations alone and incorporate factors such as systemic health, functional status, frailty, oral hygiene capability, maintenance requirements, and patient expectations. Consequently, the selection of a rehabilitation strategy should be guided by a comprehensive assessment of both clinical conditions and patient-related factors.

No single approach appears universally applicable to all clinical scenarios. Rather, successful rehabilitation depends on the ability to match the most appropriate treatment strategy to the specific anatomical, functional, and systemic characteristics of each patient. Although several augmentation-avoidance strategies also reduce surgical invasiveness, augmentation avoidance should not be regarded as synonymous with minimally invasive treatment. Rather, the overall procedural complexity varies considerably among different rehabilitation approaches and should be evaluated within the specific anatomical and clinical context of each patient. Despite the growing body of evidence supporting augmentation-avoidance approaches, further research specifically focused on older adults is needed. Future studies should place greater emphasis on patient-centered outcomes, functional measures, maintenance burden, and age-related factors that may influence the long-term sustainability of implant-supported rehabilitation.

Ultimately, the goal of contemporary geriatric implant dentistry is not to replace conventional augmentation procedures, but to identify the most appropriate rehabilitation strategy for each individual patient through careful integration of anatomical, systemic, functional, prosthetic, and patient-centered considerations.

## Figures and Tables

**Table 1:** Principal anatomical, biological, systemic, functional, and patient-related factors reported in geriatric patients undergoing implant rehabilitation.

Domain	Factor	Characteristics Commonly Reported in the Literature
Anatomical	Alveolar ridge atrophy	Reduced bone volume following long-term edentulism
Biological	Age-related bone changes	Alterations in bone remodeling and vascularization
Systemic	Cardiovascular diseases	Frequently reported chronic medical condition
Systemic	Diabetes mellitus	Common comorbidity in elderly populations
Systemic	Osteoporosis	Frequently reported condition affecting bone metabolism
Pharmacological	Polypharmacy	Concurrent use of multiple medications
Functional	Reduced manual dexterity	Difficulty performing oral hygiene procedures
Functional	Visual impairment	Reduced ability to perform self-care procedures
Cognitive	Cognitive decline	Variable impairment of cognitive functions
Frailty-related	Frailty	Reduced physiological reserve and increased vulnerability to stressors
Patient-centered	OHRQoL and treatment satisfaction	Frequently assessed patient-reported outcomes

**Table 2:** Overview of contemporary augmentation-avoidance strategies, their principal clinical applications, commonly reported outcomes, potential relevance and clinical decision-making considerations for older adults.

Rehabilitation Strategy	Main Clinical Application	Frequently Reported Outcomes	Potential Considerations in Older Adults	Clinical Decision-Making Considerations
Short and ultra-short implants	Reduced vertical bone availability in posterior maxillae and mandibles	Implant survival, prosthetic survival, marginal bone level changes, complications	May reduce the need for sinus elevation and vertical augmentation procedures	Particularly suitable when reduced surgical burden is desirable and adequate primary stability can be achieved.
Narrow-diameter implants (NDIs)	Narrow alveolar ridges with limited horizontal bone width	Implant survival, prosthetic outcomes, marginal bone loss, complications	May reduce the need for horizontal ridge augmentation and associated surgical morbidity	Appropriate for selected cases with limited horizontal bone width when biomechanical demands are compatible with implant dimensions.
Tilted implants and full-arch concepts	Edentulous arches with limited posterior bone availability	Implant and prosthetic survival, marginal bone changes, technical complications	Optimization of residual anatomy without extensive grafting procedures	Appropriate for carefully selected edentulous patients when prosthetic planning and anatomical conditions permit avoidance of extensive augmentation.
Pterygoid implants	Severe posterior maxillary atrophy	Implant survival, biological and prosthetic complications, marginal bone changes	Alternative posterior anchorage when conventional implant placement is limited	Primarily indicated in advanced maxillary atrophy but require considerable surgical expertise and careful patient selection.
Trans-sinus implants	Reduced residual bone height beneath the maxillary sinus	Implant survival, sinus-related outcomes, prosthetic outcomes	May avoid sinus augmentation procedures in selected patients	Suitable only in selected anatomical situations and when performed by experienced clinicians because of procedural complexity.
Zygomatic implants	Severe or extreme maxillary atrophy	Implant survival, prosthetic survival, surgical and biological complications	Rehabilitation of severely resorbed maxillae without extensive reconstructive surgery	Consider mainly when conventional implant placement would otherwise require extensive reconstruction; procedural complexity remains substantial.
Immediate loading protocols	Implant-supported rehabilitation requiring shortened treatment pathways	Implant survival, prosthetic outcomes, patient satisfaction	Reduced treatment duration and earlier functional restoration	Appropriate when adequate primary stability and favorable systemic and anatomical conditions support predictable immediate function.
Flapless and computer-guided surgery	Prosthetically driven implant placement and minimally invasive surgery	Surgical accuracy, implant survival, postoperative outcomes	Potential reduction in surgical invasiveness and postoperative morbidity	Best suited for appropriately selected cases with favorable anatomy and accurate digital planning.
Convergent (Hyperbolic) Neck Implants	Implant rehabilitation in healed ridges with adequate native bone, aiming to preserve peri-implant soft tissues through a transmucosal healing approach	Stable peri-implant hard and soft tissues, favorable marginal bone maintenance, high implant survival, and reduced need for second-stage surgery	May contribute to a less invasive rehabilitation pathway by simplifying soft tissue management; however, evidence specifically focused on frail geriatric patients remains limited	May facilitate soft tissue management in selected patients, although current evidence remains limited regarding specific advantages in frail older adults.
Implant-retained overdentures	Edentulous patients requiring removable implant-supported rehabilitation	Oral health-related quality of life, masticatory function, patient satisfaction, maintenance outcomes	Improved prosthesis stability with simplified hygiene and maintenance requirements	Frequently represent an appropriate option for patients with reduced manual dexterity, limited hygiene capability, or when simplified long-term maintenance is a priority.

*Clinical decision-making considerations represent interpretative observations derived from the reviewed literature and are intended to support, rather than replace, individualized clinical judgment*.

## Data Availability

The original contributions presented in this study are included in the article. Further inquiries can be directed to the corresponding author.
